# Shade tolerance in wheat is related to photosynthetic limitation and morphological and physiological acclimations

**DOI:** 10.3389/fpls.2024.1465925

**Published:** 2024-12-05

**Authors:** Yong Li, Jiarong Zhao, Hongliang Ma, Lixia Pu, Jiarui Zhang, Xiulan Huang, Hongkun Yang, Gaoqiong Fan

**Affiliations:** ^1^ State Key Laboratory of Crop Gene Exploration and Utilization in Southwest China, Ministry of Science and Technology, Chengdu, Sichuan, China; ^2^ Crop Eco-physiology and Cultivation Key Laboratory of Sichuan Province, Sichuan Agricultural University, Chengdu, Sichuan, China; ^3^ Key Laboratory of Crop Eco-Physiology & Farming System in Southwest China, Ministry of Agriculture and Rural Affairs, Chengdu, Sichuan, China

**Keywords:** shade stress, shade tolerance, leaf carboxylation efficiency, sucrose metabolism, grain yield

## Abstract

Low solar irradiance reaching the canopy due to fog and heavy haze is a significant yield-limiting factor worldwide. However, how plants adapt to shade stress and the mechanisms underlying the reduction in leaf photosynthetic capacity and grain yield remain unclear. In this study (conducted during 2018–2021), we investigated the impact of light deprivation (60%) at the pre-anthesis and post-anthesis stages on leaf carboxylation efficiency, source-to-sink relationships, sucrose metabolism, and grain yield of wheat cultivars with contrasting shade tolerance. Shade stress decreased stomatal conductance, stomatal limitation value, intrinsic water use efficiency, rubisco activity, and carboxylation efficiency of flag leaves during grain-filling, whereas intercellular CO_2_ concentration increased. These findings indicate that non-stomatal limitation reduces the net photosynthesis rate in a weak-light environment. Shade-tolerant cultivars (MM-51 and CM-39) adapted to low-light conditions via a higher leaf area of flag leaves, light interception rate, and chlorophyll *a* and *b* contents; this increased non-structural carbohydrates and sucrose contents in developing grains, ultimately decreasing yield loss by shade stress. Pre-anthesis shading resulted in a greater yield loss than post-anthesis shading because of decreased plant biomass, grain number per spike and 1,000-kernel weight. This study indicates that Rubisco-mediated non-stomatal limitation reduces *P*_N_ and sucrose content in plants exposed to low-light stress, contributing to decreased grain yield. Our study provides information on the mechanism underlying shade stress tolerance, which will help design future strategies for reducing yield loss in the context of global dimming.

## 1 Introduction

Solar irradiance provides energy for crop growth and grain filling. Globally, the amount of photosynthetically active radiation (PAR) reaching the crop canopy is unstable and has decreased by 4%–6% since the 1950s due to climate change and the rapidly increasing heavy haze and aerosol pollution (Shao et al., 2021; Wang et al., 2021b). The aerosol radiative effect in PAR spectrum weakens from -122 W/m^2^ under the clear-air condition to -80 W/m^2^ during the severe-pollution period (Wang et al., 2021c; Yuan et al., 2023). The Sichuan basin is a representative wheat production region with low canopy solar irradiance, and wheat plants experience shade stress caused by rainy and cloudy weather that occurs during the grain-filling stage of wheat. Meanwhile, wheat plants will also suffer from shade stress due to the fog and decreasing solar altitude in autumn and winter. Low solar irradiance reaching crop canopy has gradually become a key factor limiting crop yields worldwide (Wang et al., 2015; Yang et al., 2020). The grain yield of wheat under otherwise optimum conditions is sensitive to shade stress (Savin and Slafer, 1991). Shade stress affects leaf photosynthetic capacity, cellular biochemistry, and daily carbon gain (Feng et al., 2018; Gao et al., 2020a); this in turn directly affects plant biomass, grain yield, and bread making quality (Shao et al., 2020; Song et al., 2022; Wang et al., 2020b). Most cereal crops have been selected for full light conditions, making it necessary to determine those able to acclimate to low irradiance environments and the traits that drive this acclimation (Arenas-Corraliza et al., 2019). Most cereal crops are particularly sensitive to shade stress between 9:00 am to 11:00 am, 20 days after anthesis (Wu et al., 2021; Naseer et al., 2021). In the face of accelerated global industrialization, determining the stomatal and non-stomatal factors that contribute to net photosynthesis rate (*P*_N_) reduction in weak-light environments and understanding how wheat plants adapt to weak-light environments are prerequisites for ensuring food security in the context of global dimming.

The conversion of solar energy reaching crop canopy to plant biomass through photosynthesis affects the attainable yield. Shade stress decreases crop yield depending on the duration of stress, crop growth stage, and canopy light environments (Yang et al., 2020). Previous research has demonstrated that shading decreases leaf photosynthesis and hinders carbohydrate redistribution, eventually reducing yield (Naseer et al., 2021; Zhang et al., 2020). However, *P*_N_ reduction caused by shade stress occurs for various reasons. Shade stress decreases the diffusion of CO_2_ from the atmosphere to the leaves, thus decreasing *P*_N_ via stomatal limitation (Lv et al., 2020). Shading alters light-capturing chlorophyll components, electron transport fragments, and energy-transferring enzymes, which directly affect the conversion of canopy solar energy to plant biomass via nonstomatal limitation (Wang et al., 2020b; Wu et al., 2021). The decrease in light intensity reaching crop canopy undoubtedly reduces leaf photosynthesis. However, uncovering the mechanisms underlying plant adaptation to low-light environments is critical for formulating breeding and crop management targets to reduce yield loss by shade stress. Previous studies have shown that plants adapt to shade stress by decreasing the light compensation point (LCP) and regulating the functions of the photosystem II (PSII) reaction center and chloroplasts (Mu et al., 2010). In weak-light environments, plants increase the chlorophyll content of leaves to maximize light capture efficiency in the PSII reaction center (Wang et al., 2020b; Wu et al., 2021). However, they decrease the chlorophyll *a*/chlorophyll *b* ratio, improving the light absorption ability of the chloroplast and thus increasing the solar energy conversion efficiency in crop canopy (Feng et al., 2018). In field conditions, the leaves within the canopy experience a continually changing light environment because of changing solar angles and leaf position due to the wind. Photosynthesis adapted to weak-light conditions includes the regeneration of the Rubisco enzyme, opening and closing of stomata, diffusion of CO_2_, and carbohydrate metabolism (Acevedo-Siaca et al., 2020 Deans et al., 2019). Evaluating responses of stomata limitation values to changing light conditions of cultivars with contrasting shade tolerance will provide insights into the limitations of photosynthesis and the daily carbon gain in weak-light environments.

Plants adapted to low-light environments maximize the capacity of flag leaves to convert canopy solar energy into carbohydrates (Mathur et al., 2018). As the primary product of photosynthesis, sucrose is a global regulator of plant response to canopy light intensity. Shade stress decreases the sucrose content in grains due to sucrose transport and cleavage (Hu et al., 2016). Sucrose availability in developing grains depends on sucrose phosphate synthase (SPS; EC 2.4.1.14) and sucrose synthase (SuSy; EC 2.4.1.13). Previous studies have suggested shade-tolerant cultivars exhibit higher light harvesting, solar energy conversion efficiency, and sucrose content than shade-sensitive cultivars (Yang et al., 2021). The effect of shading on source-to-sink relationships and grain yield depends on the cultivar, sucrose transport and cleavage, and canopy light intensity (Yang et al., 2020). More evidence is required to establish this model in different cultivars with contrasting shade tolerance and in both grains and leaves.

Our previous study indicated that canopy solar irradiance affects the macromolecular structure of protein and starch by regulating flag leaves’ apparent quantum yield and maximum net photosynthetic rate (Yang et al., 2023). To further investigate physiological factors that contribute to net photosynthesis rate (*P*_N_) reduction by shading and understanding how wheat plants adapt to weak-light environments, four wheat cultivars with contrasting shade tolerance were light deprived (60% shading) before or post-anthesis for evaluating the changes in carboxylation efficiency, Rubisco activity, and sucrose metabolism, and grain yield to determine the factors that limit *P*_N_ during grain-filling. Our study was conducted based on the hypothesis that leaf photosynthesis of plants exposed to shade stress decreases due to Rubisco-induced non-stomatal limitations, which reduces sucrose availability in grains, ultimately decreasing plant biomass and grain yield. The second hypothesis is that shade-tolerant cultivars adapt to low-light environments and show a high grain yield through morphological and physiological acclimations. Our study provides information for uncovering the mechanisms underlying shade stress tolerance, which will be useful for designing crop management and breeding strategies to reduce yield loss in the context of global dimming.

## 2 Materials and methods

### 2.1 Experimental site and design

**Figure 1:**
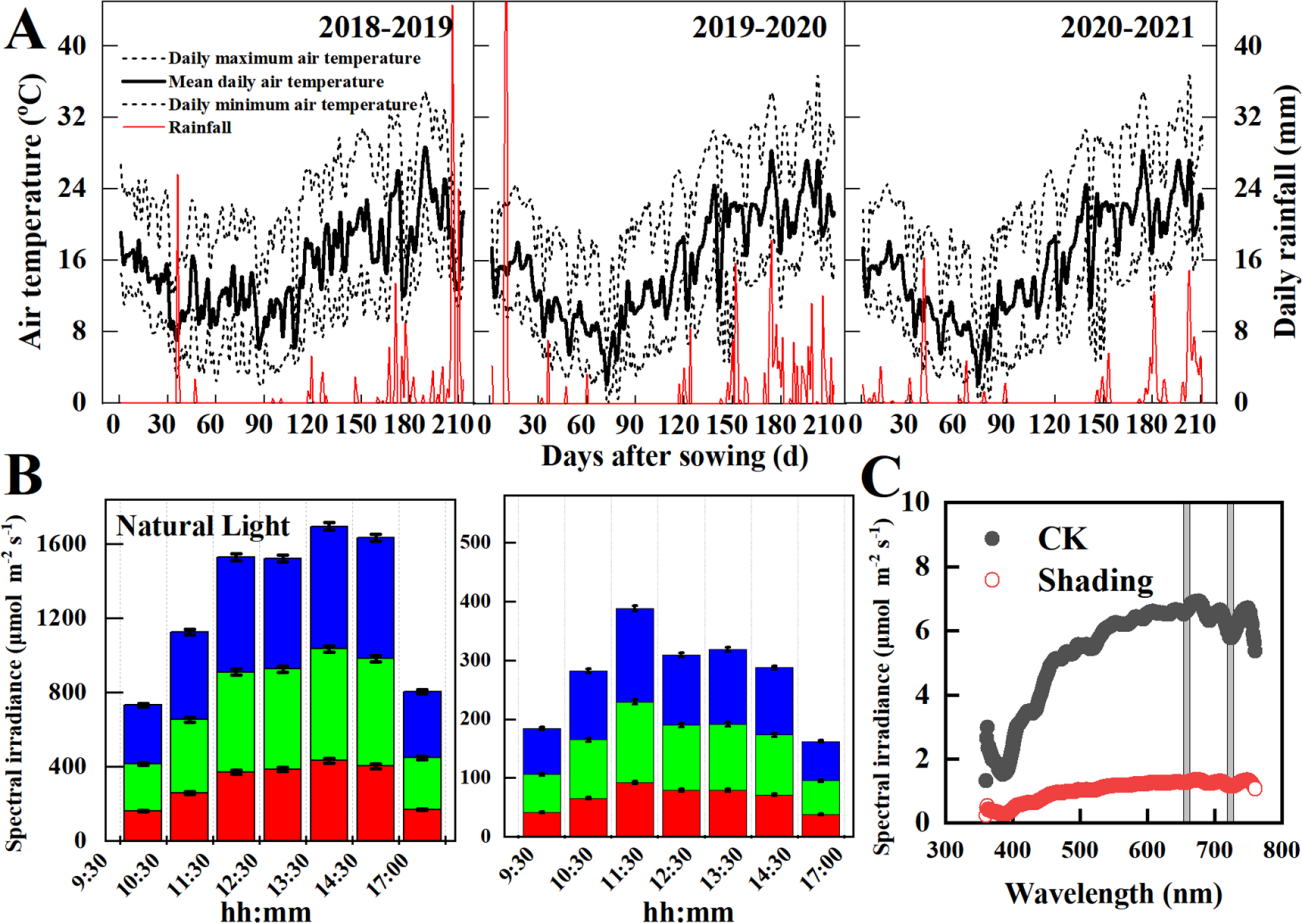
Climate conditions **(A)**, daily variations of photosynthetic active radiation **(B)**, and spectral irradiance **(C)** at the study site in the 2018-2019, 2019-2020, and 2020-2021 wheat growing seasons.

During the 2018–2019, 2019–2020, and 2020–2021 wheat growing seasons, field experiments were conducted at the Xichang experimental station (27° 90′ N, 102° 26′ E) in Southwest China. The mean air temperatures during the wheat growing season were 14.3°C, 14.2°C, and 14.2°C, respectively, and the total precipitations were 193 mm, 235 mm, and 191 mm, respectively (**Figure 1A**). According to the world reference base for soil sources (Nachtergaele et al., 2000), the soil at the experimental site is typical of eutric chromic cambisol. Soil nutrition at the 0–20 cm soil layer was enriched in the soil available K (115 mg kg^–1^), and lack in soil organic matter (15.70 g kg^–1^), Total N (1.18 g kg^–1^), and Olsen-P (14.4 mg kg^–1^). The sunshine hours during the wheat growth were 6.53 h in 2018–2019, 7.43 h in 2019–2020, and 6.97 h in 2020–2021, and the average solar radiation at noon was 1,600 μmol m**^–^** ^2^ s**^–^** ^1^.

A split**-**plot experimental design was used in this study. The design included two treatment combinations with three replications each: three shading treatments as the main plot and four cultivars with contrasting shade tolerance as subplots (Yang et al., 2023). Each plot was 4 m wide and 10 m long. Four widely grown wheat cultivars, Changmai-34 (CM-34), Chuanmai-39 (CM-39), Shumai-482 (SM-482) and Mianmai-51 (MM-51), were used as the experimental plants. The four wheat cultivars had similar plant heights (80**–**85 cm), days from wheat sowing to harvest (180–190 d) and days from sowing to anthesis (135-140 d). The CM-34 and SM-482 were considered low-light**-**sensitive cultivars compared to CM-39 and MM-51 (Yang et al., 2023). In all three cropping seasons, wheat seeds were sown by hand on Oct-30 at 250 seedlings m**^–^** ^2^, row spacing of 20 cm, and plant spacing of 10 cm. Wheat plants experience shade stress caused by rainy and cloudy weather that occurs during the grain-filling stage of wheat. Meanwhile, wheat plants will also suffer from shade stress due to the fog and decreasing solar altitude in autumn and winter. Therefore, the shading treatments were applied: CK, unshaded control; S1, shaded from the four-leaf stage (GS16) to the anthesis (GS64); S2, shaded from anthesis to maturity (GS94). Solar irradiance is estimated to be reduced by 28%–49% or even higher, calculated using the ultraviolet-visible (TUV) model (Tie et al., 2016). Therefore, shading was treated by using a black Sarlan shade cloth that decreased canopy light intensity by 60 ± 3% (**Figure 1B**) and did not affect the red and far-red ratios (*P<* 0.05; **Figure 1C**). The shading net was placed 2 m above the ground along the sides of each plot, and a 10 cm space was maintained on the north and south sides to increase ventilation and ensure minimum variation in air humidity (increased by 0.9%, not significant) and temperature (decreased by 0.2°C, not significant). Fertilizer and pest management followed local high**-**yielding practices.

### 2.2 Sampling and measurements

The flag leaves of wheat and their corresponding spikes were labelled using plastic tags listing the anthesis date. The flag leaves and their corresponding spikes were collected at 0, 7, 14, 21, 28 35 and 42 days after anthesis from 9:00 a.m. to 10:00 a.m. Separated grains and flag leaves were placed in liquid nitrogen and stored at −80°C. Half of the leaves were used for assays of Rubisco, PEPC, SuSy SPS and PEPC. The other half of the flag leaves were used for determining chlorophyll, sucrose and soluble sugar contents. Half of the developing grains were used for assays of SuSy SPS and PEPC. The other half of the grains were used for determining 1000-grain weight, sucrose and soluble sugar contents.

#### 2.2.1 Leaf morphological traits and light interception

The leaf area was recorded by measuring the leaf length and width of the flag leaves with a conversion factor of 0.75 in each plot. The leaf area index is the ratio of leaf area to land area, and the maximum value of LAI was recorded at the booting stage. The leaf mass area (LMA) is the ratio of leaf dry mass and leaf area. The fraction of light inception of the wheat canopy was measured above and below the canopy in each plot between 11:00 a.m. and 2:00 p.m. on a typical sunny day by using a quantum sensor (AccuPAR LP-80, Decagon Devices) at the stem extension, heading, and maturation stages (Zadoks et al., 1974). The fraction of the light interception at noon (*f*PARn, Equation 1) and the daily light interception (*f*PAR, Equation 2) were calculated as following equations (Du et al., 2015):


(1) $$f{\text{PAR}}_{\text{n}}\text{ = }1-\frac{I_{0}}{I_{t}}$$

(2) $$f\text{PAR = (}\frac{2f\text{PAR}n}{1+f\text{PAR}n})$$

#### 2.2.2 Gas-exchange parameters and carboxylation efficiency of flag leaves

The *P*_N_, transpiration rate (*T*_r_), stomatal conductance (*g*_S_), and intercellular CO_2_ (*C*_i_) were measured using an LI-6800 XT portable photosynthesis system (LI-COR, Lincon, NE, USA) at 7, 21 and 35 days after anthesis (DAA). Steady**-**state gas exchange parameters were recorded after the leaves were clamped for 5 min, and photosynthetic parameters were recorded at 1,200 μmol m**^–^** ^2^ s**^–^** ^1^ light intensity, 380 ± 5 μmol mol**^–^** ^1^ CO_2_, and 70% humidity between 9:00 a.m. and 11:00 a.m. All measurements were made on the central portion of the flag leaves and averaged over at least three replicates per plot.

The photosynthetic light response curve of the flag leaves was measured at anthesis between 9:00 a.m. and 11:30 a.m. The *P*_N_, *g*_S_, *C*_i_, and *T*_r_ were recorded at photosynthetic photon flux densities of 2,000; 1,800; 1,500; 1,200; 1,000; 800; 600; 400; 200; 150; 100; 50; 30; and 0 μmol m**^–^** ^2^ s**^–^** ^1^ (Arenas-Corraliza et al., 2019). These measurements were recorded at a CO_2_ concentration of 400 ± 5 μmol mol**^–^** ^1^ (maintained using CO_2_ cylinders). To quantify the responses of carboxylation efficiency (CE, Equation 3), stomatal conductance (*g*_S_, Equation 4), intrinsic water use efficiency (WUE_i_, Equation 5), stomatal limitation value (*L*s, Equation 6) to solar irradiance, a non-linear parameter estimation procedure was used to fit the relationship of these parameters with photosynthetically active radiation (PAR) according to Ye model (Ye and Yu, 2008, Ye et al., 2013, Ye et al., 2020).


(3) $$CE=\frac{P_{N}}{C_{i}}=\frac{\alpha_{2}}{C_{i}}\times \frac{1-\beta_{2}\times PAR}{1+\gamma_{2}\times PAR}\times PAR+\frac{R_{d}}{C_{i}}$$

(4) $$g_{S}=\alpha_{0}\frac{1+\beta_{0}\times PAR}{1+\gamma_{0}\times PAR}\times PAR+g_{s0}$$

(5) $$WUE_{i}=\frac{P_{N}}{g_{S}}=\frac{\alpha_{2}}{g_{S}}\times \frac{1-\beta_{2}\times PAR}{1+\gamma_{2}\times PAR}\times PAR+\frac{R_{d}}{g_{S}}$$

(6) $$L_{s}=\alpha_{1}\frac{1-\beta_{1}\times L}{1+\gamma_{1}\times PAR}\times PAR+L_{s0}$$

(7) $$L_{s-sat}=\frac{\sqrt{(\beta_{1}+\gamma_{1})/\beta_{1}}-1}{\gamma_{1}}$$

where *α*, *β*, and *γ* are fixed coefficients determined by regression analysis; *L*_s-sat_ is the stomatal limit value at saturated light intensity; *g*_S0_ and *L*_s0_ denote stomatal conductance and stomatal limit value in the dark, respectively; *R*_d_ is the dark respiration rate.

#### 2.2.3 Rubisco enzyme assays

Photosynthetic proteins were extracted using a method previously described (Carmo-Silva et al., 2017), with minor modifications. Leaf samples were homogenized in 50 mM Tris-HCl (pH 7.5), 5 mM magnesium chloride (MgCl_2_), 1 mM EDTA, 12.5% (v/v) glycerin, 10% (v/v) polyvinylpyrrolidone, and 50 mM dithiothreitol. Subsequently, the samples were centrifuged at 15,000 × g for 15 min at 4°C, and the supernatant was used to determine the activity of Rubisco at 25°C.

Rubisco activity was determined in reaction mixtures containing 50 mM of Tris-HCl (pH 8.0), 15 mM of MgCl_2_, 1 mM of EDTA, 10 mM of NaCl, 10 mM of NaHCO_3_, 5 mM of DTT, 5 mM of phosphocreatine, 5 mM of ATP, 0.12 mM of NADH, 7 units of glyceraldehyde-3-phosphate dehydrogenase (GAPHD, Sigma), 7 units of phosphoglycerate kinase (PGK, Sigma), 7 units of creatine phosphokinase (CPK, Sigma), 0.6 mM RuBP (added to tubes individually), and 5 μL of the supernatant. The activity was determined by monitoring the oxidation rate of NADH at 340 nm, assuming that two molecules of NADH were oxidized per molecule of CO_2_ fixed.

#### 2.2.4 Sucrose content and sucrose metabolic enzymes

Non-structural carbohydrates in both leaves and developing grains were extracted with 80% ethanol and quantified using the anthracene sulfuric acid method (Yang et al., 2017) and a benchmark microplate reader (Bio**-**Rad, Inc., Hercules, CA, USA). The sucrose content in both leaves and developing grains was estimated using the KOH-resorcinol method (Yang et al., 2017).

The enzyme extracts of SuSy (EC 2.4.1.13) and SPS (EC 2.4.1.14) were prepared by homogenizing leaf tissues in 5 mL of a buffer solution containing 50 mM HEPES-NaOH (pH 7.5), 10 mM MgCl_2_, 1 mM Na-EDTA, 1 mM Na-EGTA, 5% (v/v) glycerol, 0.1% (v/v) Triton X-100, 2.5 mM DTT, and 2% (w/v) PVP (Yang et al., 2017). After the homogenates were centrifuged at 15,000 × g for 20 min at 4°C, the supernatant was maintained at 4°C and assayed immediately. The SuSy reaction mixture contained 20 mM Pipes-KOH buffer (pH 6.5), 100 mM sucrose, 2 mM UDP, and 200 μL enzyme extract. The mixture was incubated in a water bath at 30°C for 30 min, and the reaction was terminated by adding 250 μL of 500 mM tricine-KOH buffer (pH 8.3). The SuSy activity was determined based on the amount of fructose produced from sucrose. The SPS reaction mixture contained 14 mM UDP-glucose (UDPG), 50 mM fructose-6-P, 10 mM MgCl_2_, and 200 μL of the extracted enzyme. The reaction mixture was incubated at 30°C for 30 min, and the reaction was terminated by adding 0.1 mL of 1 M NaOH and heating the solution for 10 min at 100°C. The sucrose generated was quantified using the KOH-resorcinol method.

The phosphoenolpyruvate carboxylase (PEPC) enzyme of leaf tissue was extracted using a mixture of 50 mM HEPES-Tris (pH 7.0), 8 mM Na-EDTA, 5% (v/v) glycerol, 0.1% (v/v) 4 mM DTT, and 2 mM PMSF (Yang et al., 2018). After the homogenates were centrifuged at 15,000 × g for 20 min at 4°C, the supernatants were maintained at 4°C and assayed immediately. The reaction mixture contained 30 mM HEPES-Tris (pH 7.5), 10 mM MgCl_2_, 0.25 mM NADH, 5 mM DTT, and 10 mM NaHCO_3_. The reaction was initiated by adding 10 U of malate dehydrogenase (MDH) and 100 μL of 30 mM phosphoenolpyruvate (PEP) to a final volume of 1.05 mL. After the reaction mixture was incubated in a water bath at 30°C for 30 min, the NADPH oxidation was measured at 340 nm. PEPC activity was expressed as mM NADPH per gram of fresh weight per hour.

#### 2.2.5 Grain-filling characteristics and structure of endosperm

Panicles were collected every 7 d (starting from day 0) between 9:00 a.m. and 11:00 a.m. after anthesis until grain maturation. The kernels from each sampling date were separated, counted, and oven-dried at 105°C for 30 min and then at 60°C for 3 d to attain a constant weight. Richard’s growth function (Equation 8) was used to assess the grain dry mass accumulation rate (Equation 9) (Wang et al., 2017):


(8) $${\text{W}}_{\left(\text{g}\right)}=\frac{{\text{W}}_{\text{max}}}{{\left(1+\text{B}{\text{e}}^{-\text{kt}}\right)}^{\frac{1}{\text{N}}}}$$

(9) $${\text{V}}_{\;(\text{g}{\text{d}}^{-1})}=\;\frac{{\text{d}}_{\text{w}}}{{\text{d}}_{\text{t}}}\;=\frac{{\text{W}}_{\text{max}}\text{kB}{\text{e}}^{-\text{kt}}}{{\left(1+\text{B}{\text{e}}^{-\text{kt}}\right)}^{\frac{\text{N}+1}{\text{N}}}}$$

where W (g) is the kernel dry weight at development time (d) and W_max_ is the 1,000-kernel weight at maturity. B, k, and N are fixed coefficients determined by sigmoid growth function.

Starch morphology in the endosperm was photographed using a Zeiss Merlin Compact scanning electron microscope (SEM, Zeiss, Oberkochen, Germany), following a previously described method (Zhou et al., 2020). The endosperm for each treatment was fixed in an aluminum foil film, and dried at 40°C for 4 h. After the samples were mounted on a metal stub covered with gold, observed, and then photographed (Gao et al., 2020b).

#### 2.2.6 Plant dry mass, grain yield and yield components

The aboveground plant dry mass was sampled from 30 consecutive wheat plants at the anthesis and maturation stages. The plant samples were separated into leaves, stems, chaff, and kernels, oven-dried at 105°C for 30 min and 70°C for 72 h, and then weighed.

Grain yield was measured by harvesting a representative plot of 4 m^2^ for each plot at crop maturity. The fertile spikes in 4 m^2^ representative plots were counted, and the grain from the spikes was threshed and air-dried. Wheat grains in each spike (grain number spike^-1^) were calculated as the number of grains obtained from 15 wheat plants divided by the number of spikes collected from those plants. The 1,000-grain weight was measured using air-dried kernels at a grain moisture content of 13.5%. Harvest index (HI) was calculated as the ratio of grain yield to total plant dry mass yield at maturity.

### 2.3 Statistical analysis

A two**-**way analysis of variance was performed using SPSS version 19.0 (SPSS Inc., Chicago, IL, USA) to analyze the data. All values were compared using least significant difference (LSD) tests (^*^ *P<* 0.05, ^**^ *P<* 0.01).

## 3 Results

### 3.1 Morphological and physiological acclimation under low-light conditions

**Figure 2:**
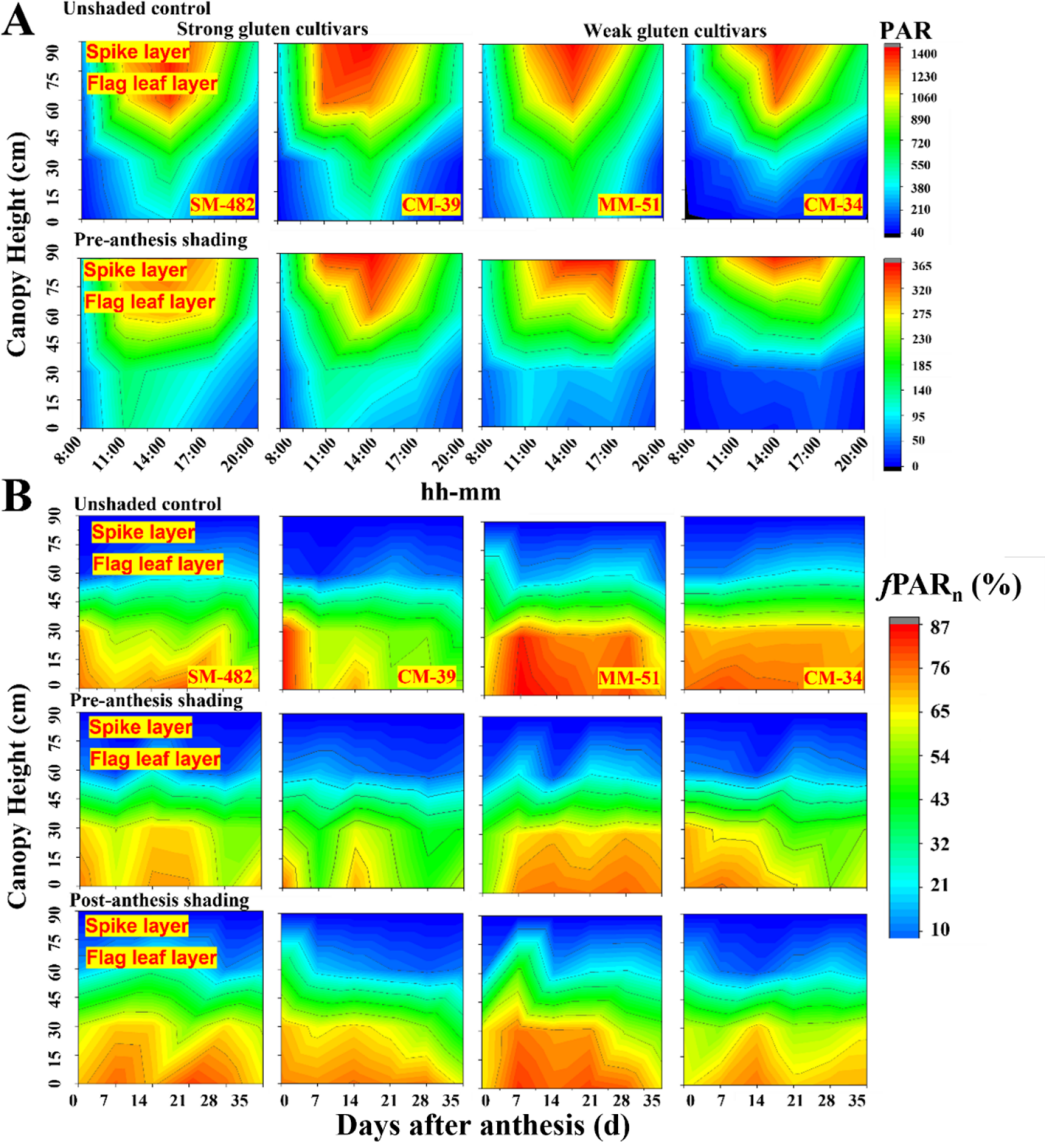
Effect of shading on daily variation in the canopy light distribution of strong and weak gluten cultivars **(A)**, and light interception rate (*f*PAR, %) with days post anthesis **(B)** in 2018-2019. MM-51 and CM-39 are shade-tolerant cultivars; CM-34 and SM-482 are shade-sensitivity cultivars.

Daily variation in the light distribution in the wheat canopy showed that the photosynthetic active radiation (PAR) increased with increasing plant height, and maximum PAR occurred around 1:00 p.m. (**Figure 2A**). Shading decreased 60 ± 3% PAR on a typical clear day, and the PAR under the bottom canopy of weak gluten cultivars (MM-51 and CM-34) showed lower values than strong cultivars (SM-482 and CM-39). The *f*PAR decreased with days after anthesis (**Figure 2B**), and a higher reduction rate was observed in weak gluten cultivars (MM-51 and CM-34) than in strong gluten cultivars (CM-39 and SM-51). Pre-anthesis shading decreased *f*PAR of SM-482, CM-39, MM-51 and CM-34 by 3.5%, 15.8%, 19.4%, and 24.1%. In contrast, post-anthesis shading increased the daily *f*PAR of SM-482, CM-39, MM-51 and CM-34 by 3.3%, 5.1%, 6.5%, and 11.3%, respectively.

Shading decreased the maximum leaf area index (LAI) and leaf mass per area (LMA), and increased the length, width, and area of the flag leaves (**Table 1**). Averaged across cultivars, the LAI and LMA of unshaded plants were 38.0% and 17.9%, respectively, higher than those of plants under post-anthesis shading and 11.5% and 1.4%, respectively, higher than those under pre-anthesis shading. In contrast, the length and area of flag leaves in plants grown under post-anthesis shading were 15.6% and 21.4%, respectively, higher than those of unshaded control. These findings indicated that shading stress decreased the total photosynthetic area of plants, and wheat plants adapt to shade stress by increasing the leaf length of flag leaves to capture more canopy light energy in weak light environments.

**Table 1:** Effects of shading and cultivars on the maximum LAI, flag leaf length and LMA values of wheat.

Cultivars	Shading	LAI_max_			Leaf length (cm)			Leaf width (cm)			Leaf area (cm^2^)			LMA (g m^-2^)		
		2018-19	2019-20	2020-21	2018-19	2019-20	2020-21	2018-19	2019-20	2020-21	2018-19	2019-20	2020-21	2018-19	2019-20	2020-21
Strong gluten cultivars																
SM-482	CK	4.92 ± 0.16 a	5.00 ± 0.24 a	5.03 ± 0.11 a	14.17 ± 0.6 b	22.0 ± 0.2 b	20.4 ± 0.3 b	1.41 ± 0.2 b	1.35 ± 0.1 c	1.31 ± 0.1 b	16.6 ± 1.1 b	20.6 ± 1.0 b	18.8 ± 0.9 b	50.4 ± 0.5 a	52.2 ± 0.3 a	51.4 ± 0.6 a
	S1	4.04 ± 0.09 b	4.10 ± 0.17 b	4.16 ± 0.08 b	14.32 ± 0.4 b	22.1 ± 0.2 b	20.2 ± 0.2 b	1.44 ± 0.1 b	1.40 ± 0.0 b	1.37 ± 0.6 b	17.5 ± 0.8 b	21.3 ± 0.4 b	19.0 ± 1.2 b	45.6 ± 0.6 a	47.2 ± 0.4 b	49.8 ± 1.3 a
	S2	3.01 ± 0.07 c	2.97 ± 0.06 c	3.03 ± 0.04 c	17.05 ± 0.6 a	25.8 ± 0.4 a	26.0 ± 0.3 a	1.79 ± 0.2 a	1.46 ± 0.1 a	1.55 ± 0.3 a	25.3 ± 0.6 a	27.3 ± 0.3 a	24.3 ± 0.7 a	33.1 ± 0.4 b	31.8 ± 0.3 c	35.0 ± 1.1 b
CM-39	CK	5.26 ± 0.10 a	5.27 ± 0.13 a	5.15 ± 0.09 a	13.91 ± 0.8 b	20.6 ± 0.4 b	19.8 ± 0.3 b	1.23 ± 0.2 b	1.53 ± 0.1 a	1.41 ± 0.2 b	14.3 ± 1.4 b	22.1 ± 1.2 a	18.0 ± 1.0 b	61.9 ± 0.5 a	60.2 ± 0.4 b	62.4 ± 0.9 a
	S1	4.22 ± 0.08 b	4.31 ± 0.11 b	4.30 ± 0.20 b	13.96 ± 0.7 b	20.5 ± 0.5 b	20.1 ± 0.3 b	1.26 ± 0.2 b	1.52 ± 0.1 a	1.39 ± 0.4 b	20.9 ± 1.5 a	21.4 ± 0.5 a	22.6 ± 1.5 a	60.1 ± 0.8 a	64.4 ± 0.4 a	63.5 ± 1.0 a
	S2	3.13 ± 0.09 c	3.01 ± 0.06 c	3.06 ± 0.15 c	17.16 ± 0.3 a	23.0 ± 0.1 a	23.3 ± 0.2 a	1.54 ± 0.3 a	1.30 ± 0.1 b	1.58 ± 0.2 a	21.9 ± 1.3 a	21.6 ± 1.0 a	22.4 ± 1.2 a	55.3 ± 0.7 b	56.5 ± 0.4 c	53.8 ± 1.1 b
Weak gluten cultivars																
MM-51	CK	5.55 ± 0.16 a	5.51 ± 0.21 a	5.54 ± 0.12 a	16.03 ± 0.3 b	22.8 ± 0.1 c	21.5 ± 0.2 b	1.62 ± 0.2 a	1.61 ± 0.0 a	1.66 ± 0.1 a	21.6 ± 0.6 c	23.9 ± 0.9 b	22.1 ± 0.4 c	48.9 ± 0.4 a	50.0 ± 0.2 a	47.2 ± 0.5 a
	S1	5.08 ± 0.04 b	5.14 ± 0.20 a	5.09 ± 0.05 b	16.17 ± 0.4 b	23.6 ± 0.7 b	21.9 ± 0.4 b	1.70 ± 0.3 a	1.62 ± 0.1 a	1.64 ± 0.3 a	25.8 ± 1.0 b	26.4 ± 0.7 a	25.4 ± 0.6 b	48.2 ± 0.6 a	50.6 ± 0.7 a	48.0 ± 1.3 a
	S2	3.27 ± 0.11 c	3.48 ± 0.14 b	3.66 ± 0.10 c	19.78 ± 0.5 a	25.1 ± 0.5 a	24.7 ± 0.2 a	1.81 ± 0.1 a	1.50 ± 0.1 b	1.67 ± 0.3 a	29.8 ± 1.3 a	26.2 ± 0.7 a	28.5 ± 0.9 a	45.3 ± 0.3 b	46.1 ± 0.7 b	40.4 ± 0.4 b
CM-34	CK	6.05 ± 0.22 a	6.08 ± 0.39 a	6.11 ± 0.08 a	15.17 ± 0.2 c	23.3 ± 0.1 c	22.2 ± 0.4 b	1.64 ± 0.2 a	1.60 ± 0.1 a	1.54 ± 0.2 a	20.7 ± 0.6 c	25.4 ± 0.5 c	21.1 ± 0.7 c	51.5 ± 0.5 a	50.7 ± 0.6 b	49.7 ± 0.6 a
	S1	5.84 ± 0.13 b	5.90 ± 0.51 a	5.82 ± 0.11 b	15.88 ± 0.3 b	25.1 ± 0.4 b	22.5 ± 0.3 b	1.69 ± 0.1 a	1.58 ± 0.1 a	1.51 ± 0.5 a	25.1 ± 1.1 b	26.3 ± 0.8 b	23.9 ± 0.6 b	50.9 ± 0.3 a	52.8 ± 0.7 a	50.1 ± 1.1 a
	S2	4.06 ± 0.08 c	4.11 ± 0.22 b	4.27 ± 0.09 c	20.03 ± 0.5 a	26.1 ± 0.4 a	25.8 ± 0.2 a	1.81 ± 0.2 a	1.57 ± 0.0 a	1.55 ± 0.4 a	30.1 ± 1.2 a	28.4 ± 1.4 a	29.5 ± 1.4 a	40.2 ± 0.4 b	41.3 ± 0.4 c	42.8 ± 0.9 b
Source of variance																
Cultivars (C)		44 ^**^	51 ^**^	47 ^**^	33 ^**^	38 ^**^	31 ^**^	14 ^**^	16 ^**^	15 ^**^	72 ^**^	68 ^**^	70 ^**^	1982 ^**^	2053 ^**^	1878 ^**^
Shading (S)		1855 ^**^	2031 ^**^	2102 ^**^	46 ^**^	43 ^**^	46 ^**^	6 ^**^	4 ^*^	5 ^**^	33 ^**^	38 ^**^	36 ^**^	954 ^**^	826 ^**^	911 ^**^
C×S		13 ^**^	7 ^**^	6 ^**^	5 ^**^	3 ^*^	5 ^**^	6 ^**^	4 ^**^	5 ^**^	15 ^**^	13 ^**^	16 ^**^	511 ^**^	503 ^**^	525 ^**^

CK, no shading; S1, pre-anthesis shading; S2, post-anthesis shading. Different letters indicate statistically significant differences at the levels of 0.05. ^∗^ *P*< 0.05; ^∗∗^ *P*< 0.01.

### 3.2 Leaf photosynthesis, stomatal conductance, and stomatal limitation values

**Figure 3:**
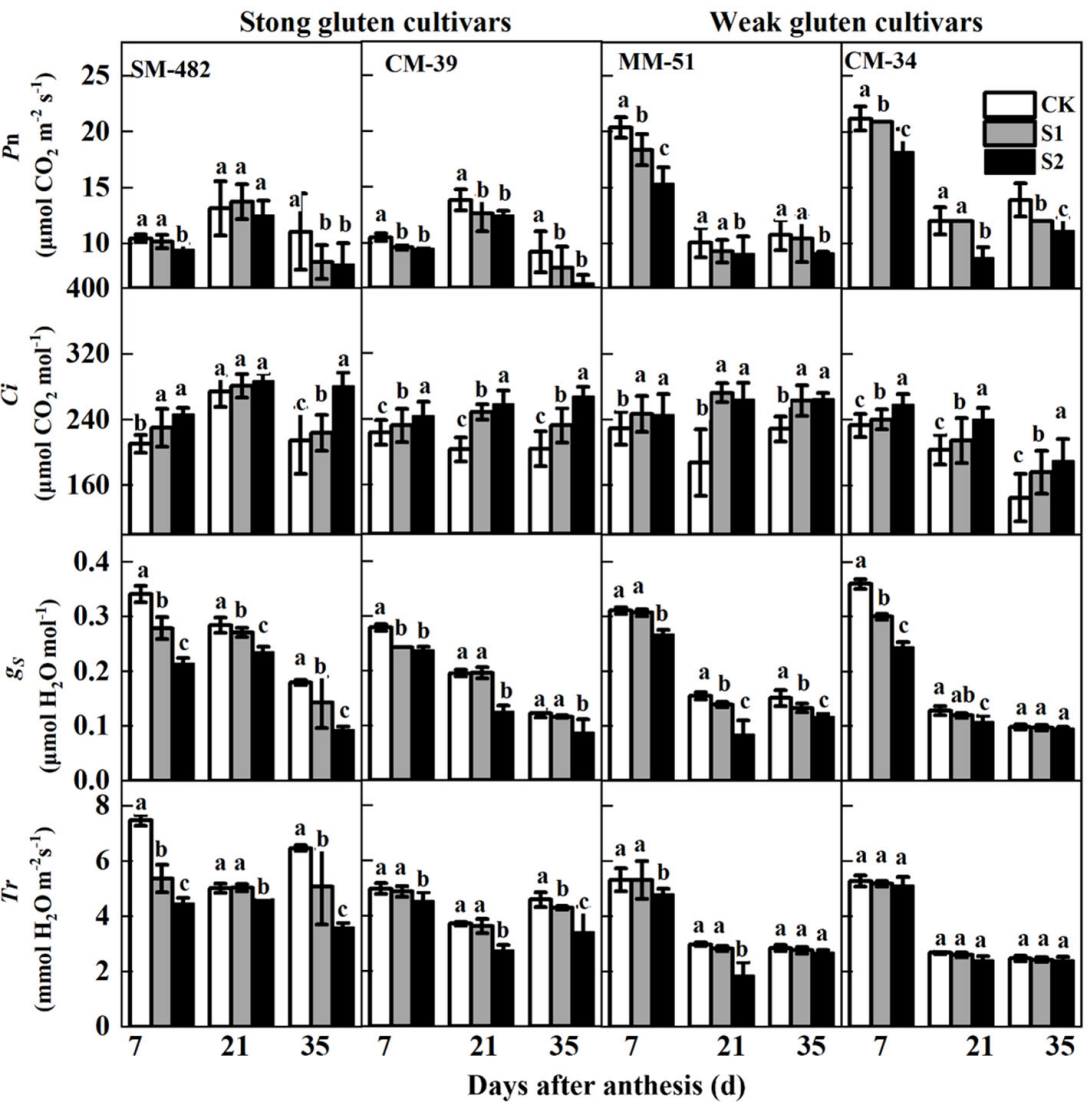
Effects of shading on the gas exchange parameters of wheat flag leaves with contrasting shade tolerance in 2018-2019. *P*_N_, *C*_i_, g_S_, and *T*r denote net photosynthesis rate, intercellular CO_2_ concentration, stomatal conductance, and transpiration rate. CK, no shading; S1, pre-anathesis shading; S2, post-anathesis shading. MM-51 and CM-39 are shade-tolerant cultivars; CM-34 and SM-482 are shade-sensitive cultivars. Data expressed as mean ± standard error (n = 3), and different letters indicate significance at 0.05 levels.

Both pre- and post-anthesis shading decreased the *P*_N_ of both strong and weak gluten cultivars (**Figure 3**). The impact of post-anthesis shading on the *P*_N_ of flag leaves was much greater than that of pre-anthesis shading treatment. Pre-anthesis shading decreased the *P*_N_ of SM-482, CM-39, MM-51, and CM-34 by 7.4%, 4.8%, 14.6%, and 30.2%, respectively, compared with those in the no-shading plots. However, post-anthesis shading decreased the *P*_N_ of SM-482, CM-39, MM-51, and CM-34 by 19.4%, 15.7%, 29.2%, and 33.0%, respectively, compared with those in unshaded control plots. These results showed that weak gluten cultivars are more sensitive to shade stress. Therefore, MM-51 and CM-34 can be classified as shade-sensitive cultivars, whereas SM-482 and CM-39 are considered as shade-tolerant.

Shading treatments increased *C*_i_ but decreased *g*_S_ and *T*_r_. The *C*_i_ of plants grown without shading was 19.6% and 28.5% lower than that of plants shaded before and after anthesis, respectively. Averaged across cultivars and sampling dates, pre-anthesis shading decreased the *g*_S_ and *T*_r_ of flag leaves by 15.3% and 14.4%, respectively, compared with those of plants in the no-shading plots. Post-anthesis shading decreased the *g*_S_ and *T*_r_ by 34.1% and 26.3%, respectively, compared with plants in the no-shading plots.

**Figure 4:**
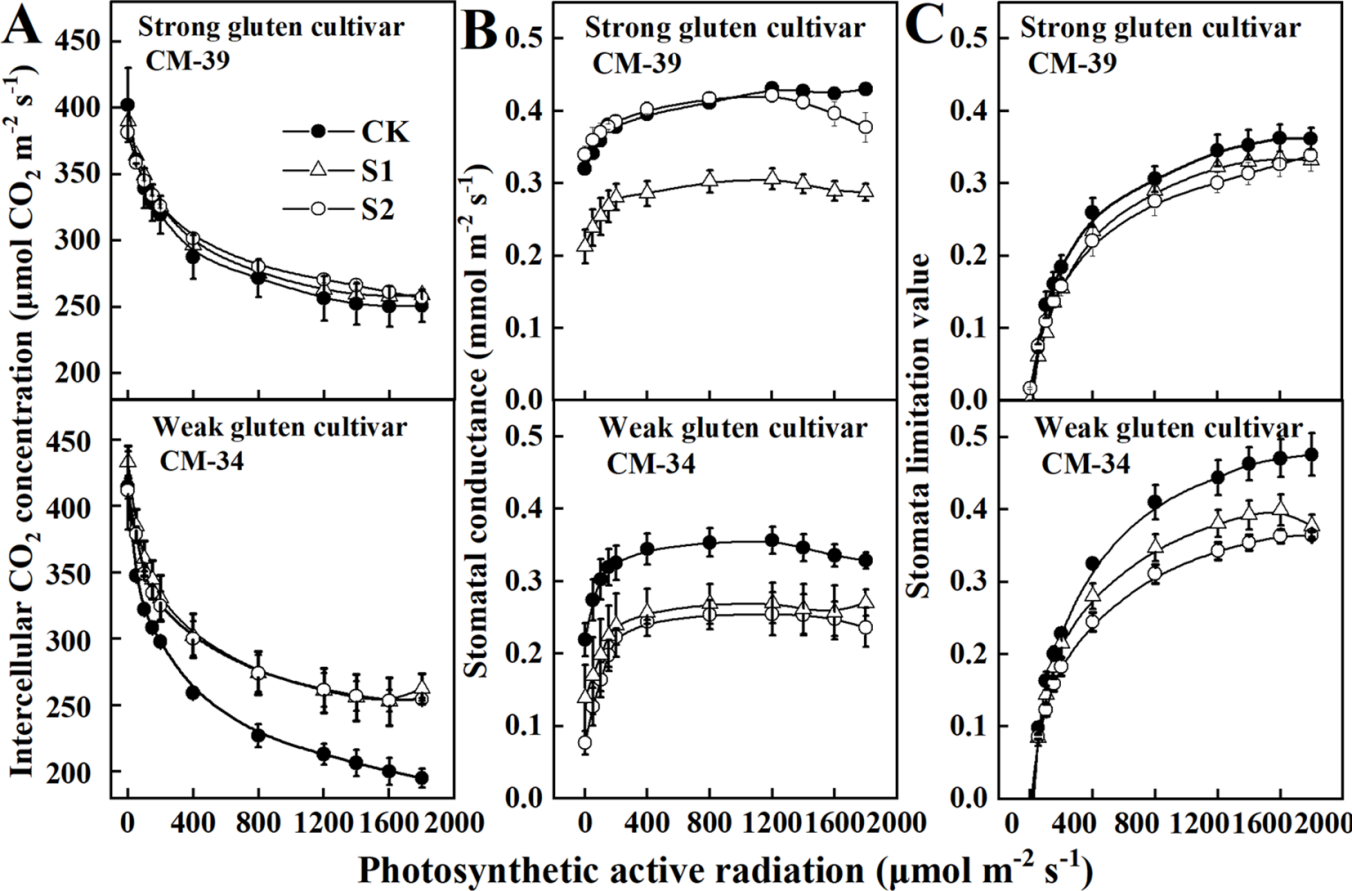
Responses of intercellular CO_2_ concentration (*C*_i_) **(A)**, stomatal conductance (*g*_S_), **(B)** and stomal limitation value (*L*_S_) **(C)** to the photosynthetic active radiation in shade tolerance (CM-39) and sensitive (CM-34) and cultivars in 2018-2019. Values are expressed as mean ± standard error (n = 3). A non-linear parameter estimation procedure was used to fit the relationship of these parameters to photosynthetically active radiation (PAR) according to the Ye model (Ye and Yu, 2008, Ye et al., 2013, Ye et al., 2020).

The responses of stomatal limitation value and stomatal conductance to canopy light intensity were evaluated to determine the physiological limitation factors that contribute to *P*_N_ reduction by shading treatments (**Figure 4**). The *C*_i_ decreased with increasing light intensity, whereas *g*_S_ and *L*s increased with increasing light intensity, allowing wheat plants to maximize CO_2_ diffusion efficiency in a shaded environment. The *g*_S_ and *L*s of plants grown under shaded conditions were lower than those of unshaded control plants. The *g*_S_ decreased more rapidly when the canopy light intensity was lower than 400 μmol m^-2^ s^-1^. However, the responses of *L*s and *g*_S_ to canopy light intensity differed between the strong and weak gluten cultivars. The *L*s and *g*_S_ of the weak gluten cultivar CM-34 (0.0006) decreased more rapidly than those of the strong gluten cultivar CM-39 (0.0005), confirming that weak gluten cultivars are more sensitive to shade stress.

### 3.3 Rubisco activity and carboxylation efficiency

**Figure 5:**
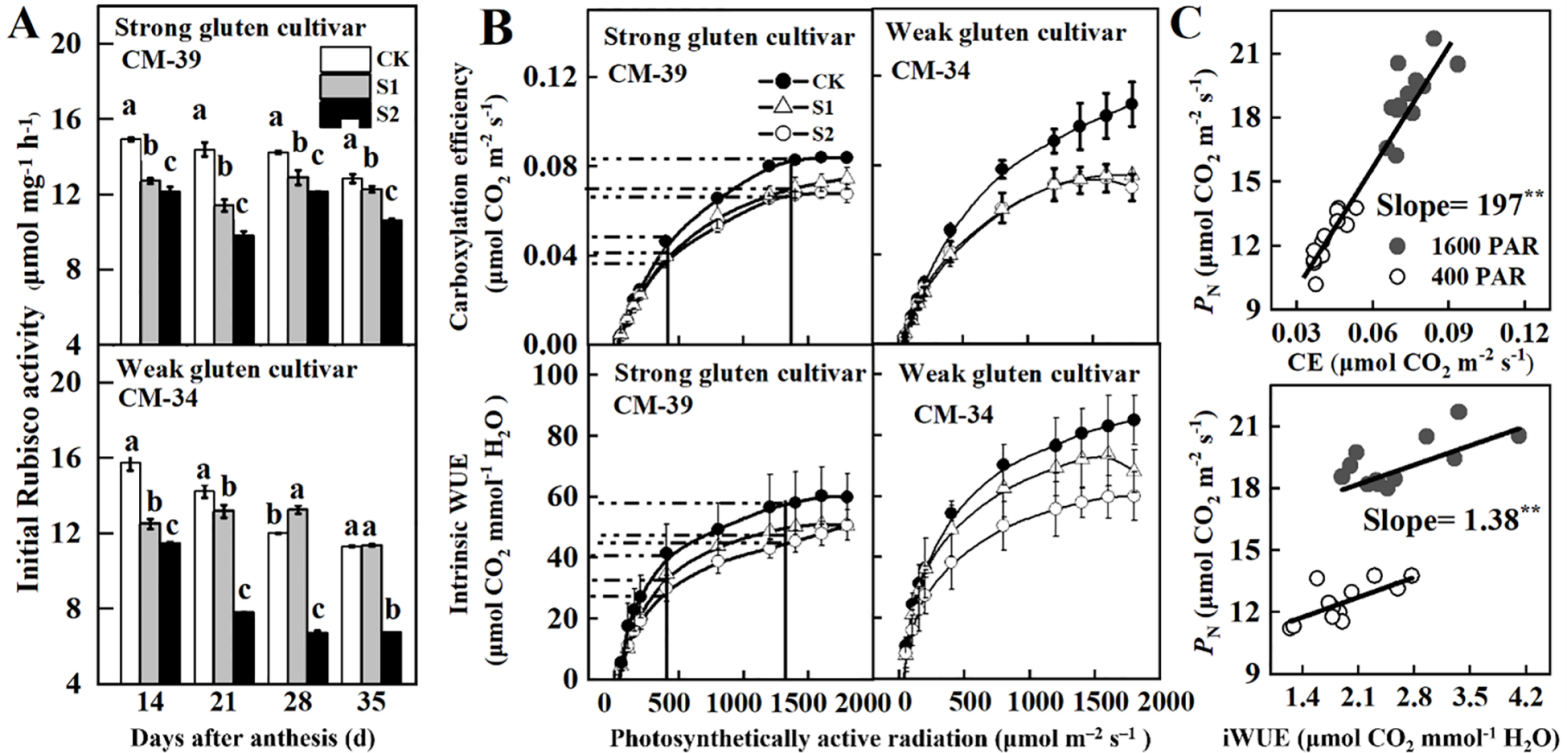
Effects of shading on initial Rubisco activity **(A)**, carboxylation efficiency (CE), intrinsic water use efficiency (WUE) **(B)**, and their relationships with *P*_N_ of wheat flag leaves in 2018-2019 **(C)**. CK, control, S1, pre-enthesis shading; S2, post-anthesis; CM-39 is a shade-tolerant cultivar; CM-34 is a shade-sensitive cultivar. Values are expressed as mean ± standard error (n = 3) and different letters indicate significance at 0.05 levels.

Rubisco activity decreased with DAA (**Figure 5**). Shading treatments decreased Rubisco activity in both shade-sensitive (CM-34) and shade-tolerant (CM-39) cultivars. The Rubisco activity in the shade-tolerant cultivar (CM-39) was higher than that in the shade-sensitive cultivar (CM-34). Under shaded conditions, the amplitude of Rubisco activity reduction also decreased with DAA.

The CE and WUE_i_ increased with increasing canopy light intensity (**Figure 5A**). Averaged across cultivars, the maximum CE and WUE_i_ of the plants grown under no-shading conditions were 57.0% and 59.5%, respectively, higher than those post-anthesis shading, and 20.3% and 55.3%, respectively, higher than those of plants under pre-anthesis shading (**Figure 5B**). In unshaded plots, the weak gluten cultivar exhibited significantly higher photosynthetic efficiency (CE) and intrinsic water use efficiency (WUE_i_) than strong gluten cultivars. However, shading stress led to greater reduction rates in CE and WUE_i_ for CM-34 than that of CM-39. Both CE (slope = 197; *R* ^2^ = 0.93^**^) and WUE_i_ (slope = 1.38; *R* ^2^ = 0.51^*^) increased linearly with *P*_N_ in both high (1,600 μmol·m^-2^·s^-1^) and low (400 μmol·m^-2^·s^-1^) light environments (**Figure 5C**).

**Figure 6:**
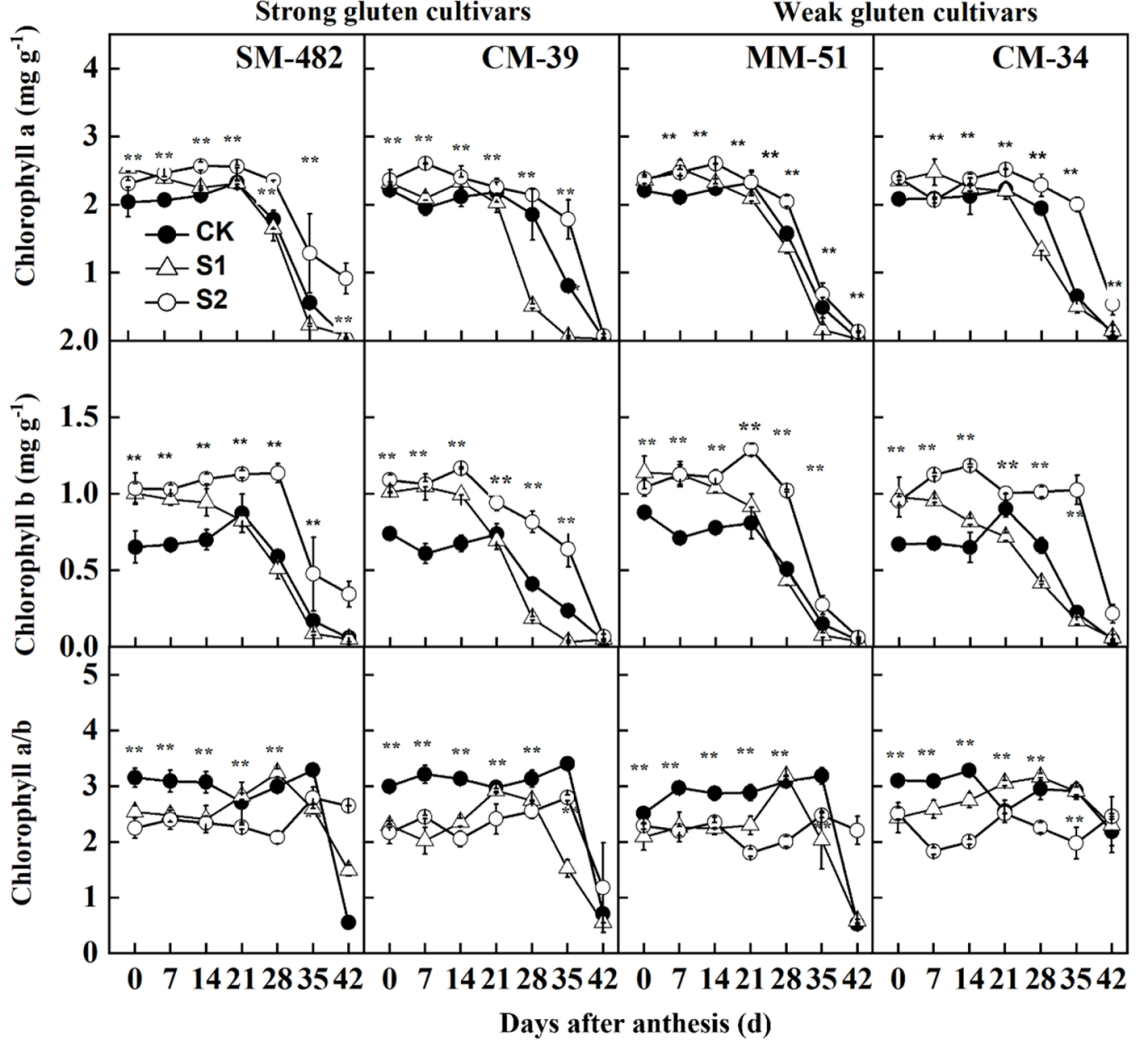
Effect of shading on chlorophyll a, chlorophyll b, and chlorophyll a/b in flag leaves of shade tolerant and shade sensitive cultivars in 2018-2019. CK, no shading; S1, pre-anathesis shading; S2, post-anathesis shading. MM-51 and CM-39 are shade-tolerant cultivars; CM-34 and SM-482 are shadesensitive cultivars. Values are expressed as mean ± standard error (n = 3).

The contents of chlorophyll *a* and chlorophyll *b* decreased sharply at 20 DAA (**Figure 6**). Shading treatments increased chlorophyll *a* and *b* contents but decreased the chlorophyll *a/*chlorophyll *b* ratio, allowing plants to use solar energy more efficiently. Averaged across cultivars and sampling dates, the chlorophyll *a* and *b* contents of plants treated with post-anthesis shading were 14.7% and 57.2%, respectively, higher than those of plants grown under no-shading conditions.

### 3.4 Sucrose content and sucrose metabolic enzymes

**Figure 7:**
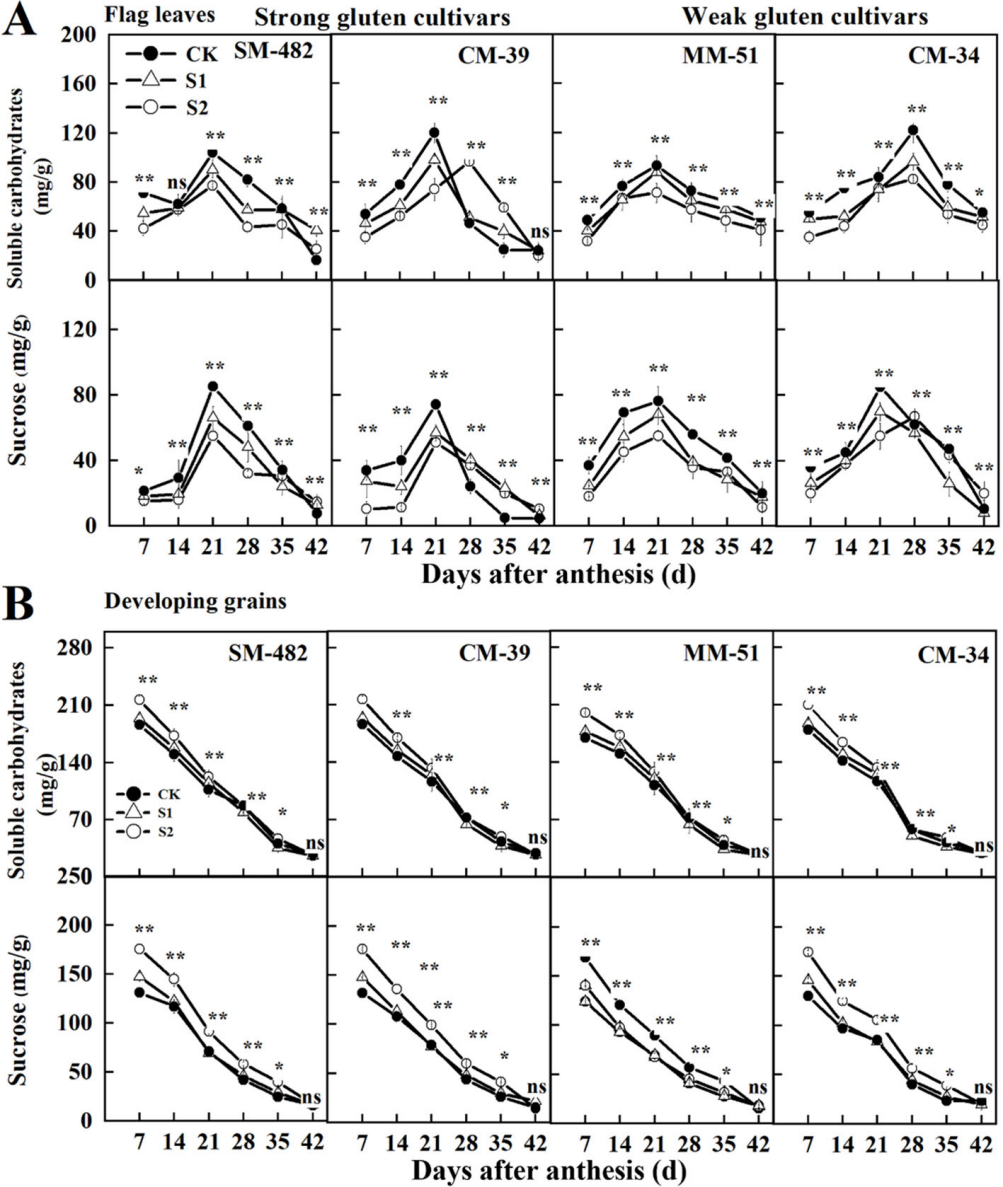
Effects of shading on soluble carbohydrates and sucrose content in wheat flag leaves **(A)** and developing grains **(B)** in 2018-2019. CK, no shading; S1, pre-anathesis shading; S2, post-anathesis shading. MM-51 and CM-39 are shade-tolerant cultivars; CM-34 and SM-482 are shade-sensitive cultivars;. Values are expressed as mean ± standard error (n = 3). ^∗^ *P<* 0.05; ^∗∗^ *P<* 0.01.

To further analyze the impact of pre- and post-anthesis shading on carbohydrate availability in both source and sink organs, we measured the non-structural carbohydrates and sucrose contents in both flag leaves and grains. The non-structural carbohydrate and sucrose contents in the flag leaves peaked at 21 DAA, whereas these values in developing grains declined with DAA (**Figure 7**). Shading treatments decreased the non-structural carbohydrate and sucrose contents in flag leaves and developing grains. The sucrose and non-structural carbohydrate contents in the developing grains of plants grown under no-shading conditions were 15.1% and 10.6%, respectively, lower than those in the developing grains of plants shaded before anthesis, and 20.9% and 8.0%, respectively, lower than those in the developing grains of plants shaded post-anthesis.

**Figure 8:**
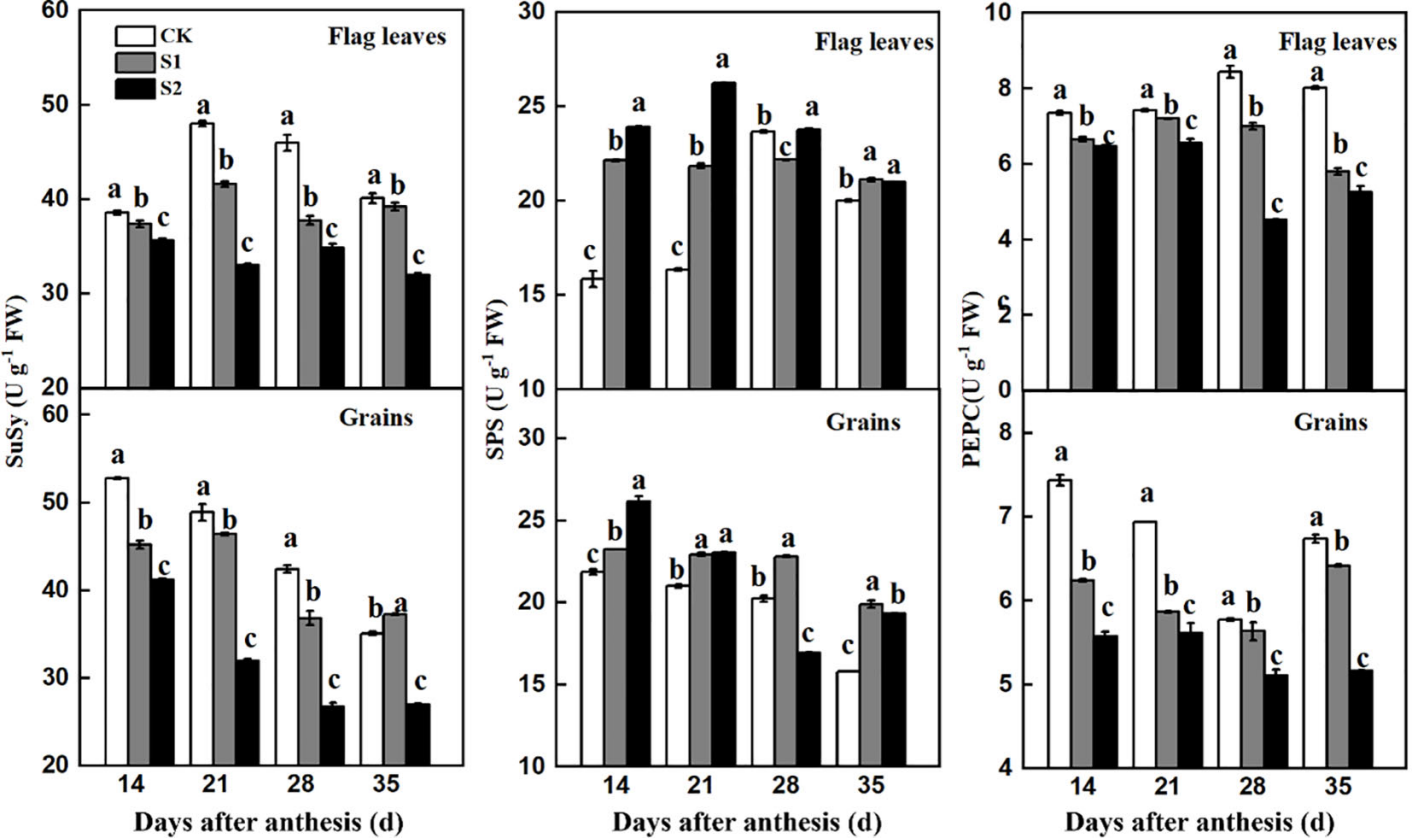
Effects of shading on SuSy, SPS, and PEPC activities in flag leaves and developing grains of shade-sensitive cultivars (CM-34) in 2018-2019. CK, no shading; S1, pre-anathesis shading; S2, post-anathesis shading. Values are expressed as mean ± standard error (n = 3) and different letters indicate significance at 0.05 levels.

The activities of SuSy and PEPC in flag leaves and developing grains in the no-shading plots were higher than those in the shaded before and after anthesis (**Figure 8**). In contrast, shading increased SPS activity in both the flag leaves and developing grains. These results confirmed that both pre- and post-anthesis shading stress decreased sucrose availability in developing grains because of decreased leaf photosynthetic carbon assimilation and sucrose metabolism.

### 3.5 Grain-filling characteristics and morphological traits of the endosperm

**Figure 9:**
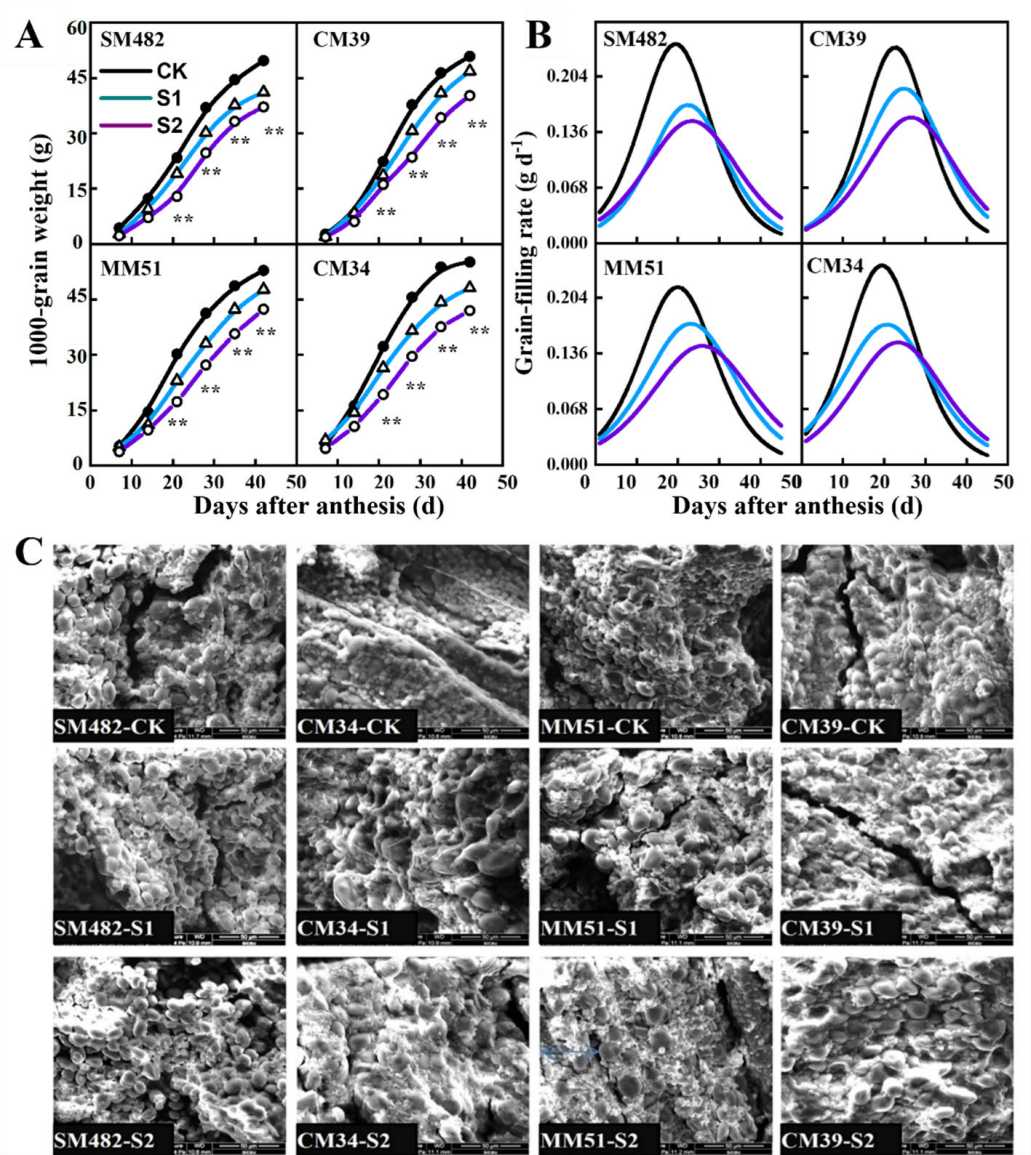
Effects of shading on the 1000-kernel weight **(A)** grain-filling rate **(B)** and the morphological characteristics of embryonic starch of cultivars with contrasting shade tolerance **(C)**. CK, no shading; S1, pre-anathesis shading; S2, post-anathesis shading. MM-51 and CM-39 are shade-tolerant cultivars; CM-34 and SM-482 are shade-sensitive cultivars. Values are expressed as mean ± standard error (n = 3). The grain-filling rate was estimated by using the second derivative of the sigmoid growth function.

Rapid grain-filling was observed between days 12 and 19, beginning at 10.8–13.8 DAA and terminating at 24.8–29.7 DAA (**Figure 9A**). Pre- and post-anthesis shading decreased the maximum grain-filling rate and duration of the rapid grain-filling period (**Figure 9B**). At maturity, pre- and post-anthesis shading decreased the 1,000-kernel weight of the crops by 6.8% and 33.3%, respectively, compared with that of the crops grown in the no-shading plots.

The morphological characteristics of embryonic starch were analyzed using a scanning electron microscope (**Figure 9C**). At maturity, starch granules and storage proteins fill the whole endosperm, and the starch granules exhibit spherical, oval, and polygonal granule structures. Shaded conditions decreased the uniformity in the morphology of starch granules. Small starch granules and storage proteins surrounded the larger starch granules. Shaded conditions also increased the amount of storage protein, the size of large starch granules (>10 μm), and the number of small starch granules. However, the size of small starch granules (<10 μm) decreased.

### 3.6 Biomass, harvest index, grain yield and yield components

**Figure 10:**
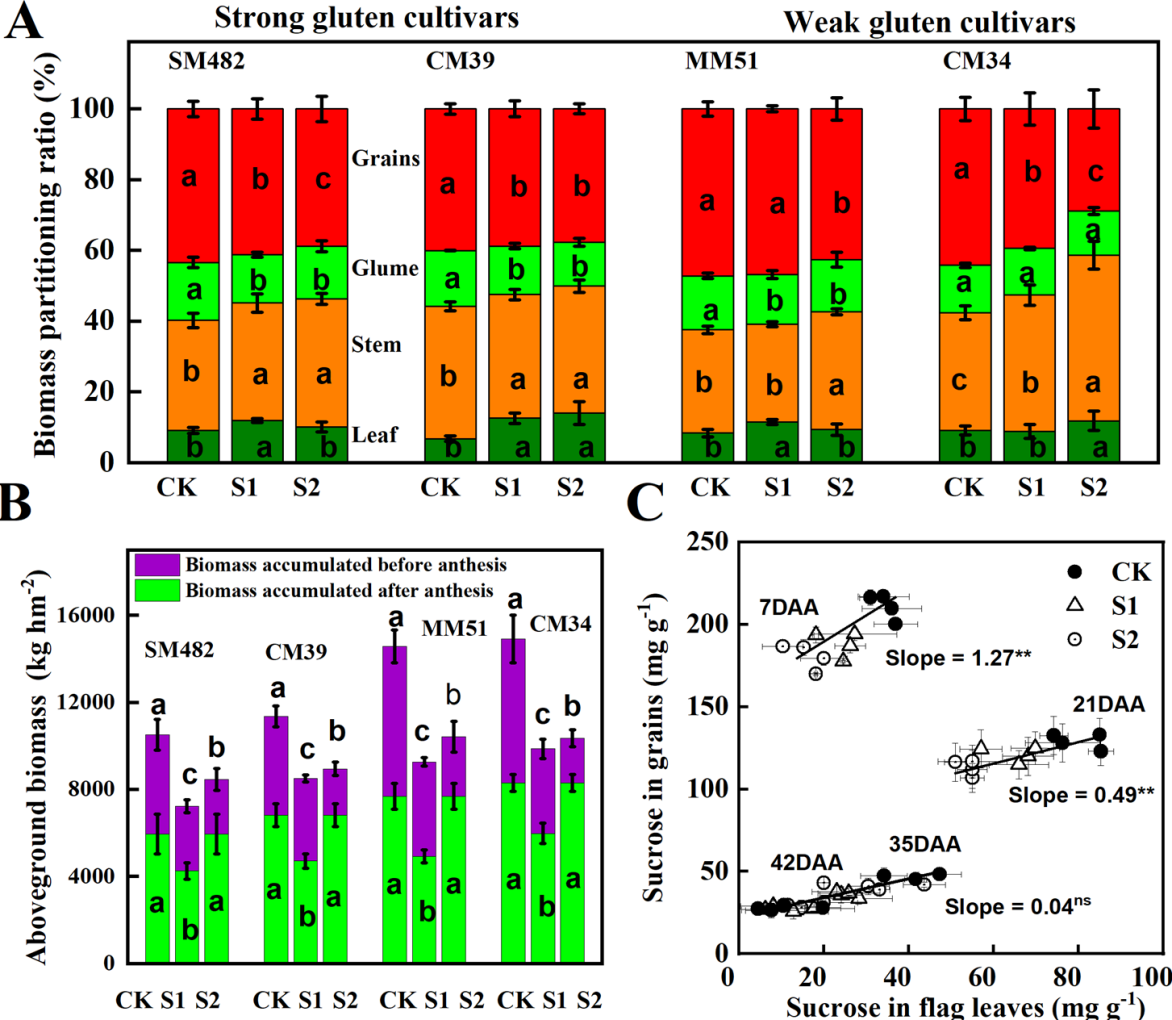
Effect of shading and cultivars on aboveground plant biomass **(A)**, biomass accumulated before and after anthesis **(B)**, and the relationship between sucrose content in flag leaves and developing grains **(C)**. CK, no shading; S1, pre-anathesis shading; S2, post-anathesis shading. MM-51 and CM-39 are shade-tolerant cultivars. CM-34 and SM-482 are shade-sensitive cultivars. Values are expressed as mean ± standard error (n = 3) and different letters indicate significance at 0.05 levels. The grain-filling rate was estimated by using the second derivative of the sigmoid growth function.

Aboveground plant biomass of SM-482, CM-39, MM-51 and CM-34 in the no-shading plots were 35.9%, 25.4%, 31.4% and 35.5%, respectively, higher than those of plants shaded before anthesis, and it was 5.6%, 3.7%, 2.1% and 1.4%, respectively, higher than those of plants subjected to post-anthesis shading (**Table 2**). Pre-anthesis shading decreased aboveground plant biomass mainly by decreasing plant biomass accumulation before anthesis, and post-anthesis shading decreased aboveground plant biomass mainly by decreasing plant biomass accumulation after anthesis (**Figure 10**). Although the plant biomass of the weak gluten cultivar was notably higher than those of strong gluten cultivars in unshaded control plots, shading stress resulted in a higher reduction rate in aboveground plant biomass for weak gluten cultivars than that of strong gluten cultivars.

**Table 2:** Effects of shading and cultivars on the fraction of light interception (*f*PAR), biomass yield, and radiation use efficiency (RUE).

Cultivars	Shading	*f*PAR at 25 DAA (%)			Biomass yield (t hm^-1^)		
		2018-19	2019-20	2020-21	2018-19	2019-20	2020-21
Strong gluten cultivars							
SM-482	CK	77.3 ± 0.6 a	72.6 ± 0.9 a	76.2 ± 0.8 a	13.1 ± 0.35 a	14.5 ± 0.21 a	15.1 ± 0.08 a
	S1	71.9 ± 0.5 c	60.6 ± 0.6 c	58.0 ± 0.6 c	8.8 ± 0.38 b	9.5 ± 0.57 c	9.0 ± 0.37 c
	S2	73.2 ± 0.4 b	68.8 ± 0.5 b	72.6 ± 0.6 b	12.7 ± 0.47 a	13.2 ± 0.26 b	14.4 ± 0.21 b
CM-39	CK	82.6± 0.5 a	77.9 ± 0.6 a	80.1 ± 0.5 a	12.7 ± 0.18 a	13.5 ± 0.00 a	11.9 ± 0.09 a
	S1	72.8 ± 0.2 c	65.4 ± 0.6 c	55.9 ± 0.6 c	8.5 ± 0.19 b	9.6 ± 0.31 b	10.2 ± 0.02 b
	S2	74.7 ± 0.7 b	73.0 ± 0.9 b	77.2 ± 0.6 b	12.2 ± 0.95 a	13.2 ± 0.29 a	11.3 ± 0.07 a
Weak gluten cultivars							
MM-51	CK	77.3 ± 0.5 a	75.4 ± 0.6 a	81.2 ± 0.7 a	17.3 ± 0.25 a	17.5 ± 0.21 a	16.4 ± 0.22 a
	S1	70.5 ± 0.3 b	73.9 ± 0.8 b	81.5 ± 0.5 a	11.2 ± 0.55 b	12.0 ± 0.21 b	11.9 ± 0.13 b
	S2	77.5 ± 0.4 a	73.7 ± 0.7 b	74.7 ± 0.5 b	16.7 ± 0.95 a	17.2 ± 0.36 a	16.2 ± 0.07 a
CM-34	CK	82.6 ± 0.5 a	82.3 ± 0.7 a	81.9 ± 0.6 a	17.6 ± 0.45 a	18.4 ± 0.13 a	16.4 ± 0.30 a
	S1	59.6 ± 0.9 c	60.5 ± 0.5 c	59.0 ± 0.4 c	10.6 ± 0.34 b	11.2 ± 0.11 c	11.4 ± 0.07 b
	S2	70.3 ± 0.6 b	66.2 ± 0.5 b	72.7 ± 0.4 b	16.7 ± 0.47 a	17.6 ± 0.12 b	15.8 ± 0.20 a
Source of variance							
Cultivars (C)		245 ^**^	257 ^**^	118 ^**^	53 ^**^	26 ^**^	127 ^**^
Shading (S)		205 ^**^	359 ^**^	209 ^**^	511 ^**^	488 ^**^	437 ^**^
C×S		47 ^**^	66 ^**^	11 ^**^	30 ^**^	53 ^**^	66 ^**^

Different letters indicate statistically significant differences at the levels of 0.05. ^∗^ *P*< 0.05; ^∗∗^ *P*< 0.01.

The highest grain yield of plants subjected to pre-anthesis shading, 3.32 **t** ha^-1^, was recorded in MM-51, followed by CM-34, CM-39, and SM-482. The grain yield of SM-482, CM-39, MM-51 and CM-34 in the no-shading plots were 55.8%, 57.1%, 57.2% and 60.7%, respectively, higher than those of plants shaded before anthesis, and it was 15.1%, 18.0%, 15.9% and 16.9%, respectively, higher than those of plants shaded after anthesis (**Table 2**). The number of fertile spikes, grain number per spike, and 1,000-grain weight of plants grown without shading were 5.0%, 9.9%, and 2.6%, respectively, higher than those of plants shaded before anthesis and 22.7%, 32.2%, and 24.3%, respectively, higher than those of plants shaded after anthesis (**Table 3**). Dominance analysis showed that the pre-anthesis shading decreased grain yield mainly by decreasing the grain number per spike and 1,000-kernel weight, whereas post-anthesis shading decreased grain yield mainly by decreasing the 1,000-kernel weight. The yield loss from shading before anthesis (2.86 **t** hm^-2^) was higher than that from shaded after anthesis (2.54 **t** hm^-2^), indicating that wheat grain yield is more limited by source intensity than sink capacity. The sucrose content in flag leaves was positively related to the sucrose content in developing grains, and the slope observed at 7 DAA was higher than that observed at 21, 35, and 42 DAA (**Figure 10C**), confirmed an increasing source limitation after anthesis under shaded environments.

**Table 3:** Effects of shading and cultivars on yield components and harvest index from no shading (CK), pre-anthesis (S1) and post-anthesis shading (S2).

Cultivars	Shading	Fertile spikes (m^-2^)			Grain number per spike^-1^			1000-kernel weight (g)			Grain Yield (t hm^-2^)			Harvest index		
		2018-19	2019-20	2020-21	2018-19	2019-20	2020-21	2018-19	2019-20	2020-21	2018-19	2019-20	2020-21	2018-19	2019-20	2020-21
Strong gluten cultivars																
SM-482	CK	315 ± 7 a	317 ± 6 a	303 ± 13 a	34.1 ± 2.4 a	35.2 ± 1.8 a	34.8 ± 1.7 a	51.0 ± 1.5 a	53.3 ± 1.8 a	44.1 ± 0.6 a	5.30 ± 0.10 a	5.85 ± 0.05 a	4.65 ± 0.05 a	0.41 a	0.39 a	0.39 a
	S1	295 ± 8 b	297 ± 8 b	268 ± 5 b	21.8 ± 2.8 b	22.4 ± 2.7 b	27.0 ± 1.0 b	32.9 ± 0.2 c	37.5 ± 0.7 c	32.3 ± 0.6 c	2.26 ± 0.14 c	2.31 ± 0.07 c	2.34 ± 0.05 c	0.24 c	0.23 c	0.23 c
	S2	314 ± 8 a	316 ± 6 a	305 ± 7 a	33.9 ± 1.6 a	34.7 ± 1.8 a	33.6 ± 1.7 a	42.5 ± 2.0 b	45.1 ± 1.7 b	39.5 ± 1.2 b	4.44 ± 0.05 b	4.91 ± 0.04 b	4.05 ± 0.05 b	0.37 b	0.34 b	0.36 b
CM-39	CK	315 ± 5 a	316 ± 3 a	334 ± 7 a	40.6 ± 2.6 a	40.7 ± 2.9 a	37.5 ± 0.2 a	44.8 ± 0.7 a	50.5 ± 0.5 a	50.5 ± 0.8 a	6.02 ± 0.08 a	6.22 ± 0.13 a	6.43 ± 0.06 a	0.45 a	0.42 a	0.42 a
	S1	292 ± 6 b	294 ± 7 b	290 ± 10 b	28.5 ± 3.0 b	29.3 ± 2.5 b	24.7 ± 1.6 b	33.0 ± 0.9 c	33.7 ± 1.1 c	35.2 ± 1.5 c	2.59 ± 0.02 c	2.89 ± 0.06 c	2.52 ± 0.09 c	0.29 c	0.27 c	0.28 c
	S2	313 ± 4 a	315 ± 6 a	330 ± 8 a	40.5 ± 3.1 a	39.2 ± 3.4 a	36.8 ± 1.2 a	37.4 ± 2.1 b	41.9 ± 2.0 b	43.7 ± 0.7 b	4.63 ± 0.05 b	5.39 ± 0.16 b	5.31 ± 0.07 b	0.39 b	0.36 b	0.37 b
Weak gluten cultivars																
MM-51	CK	322 ± 4 a	324 ± 5 a	319 ± 7 a	43.3 ± 1.6 a	43.8 ± 1.7 a	47.8 ± 1.4 a	54.9 ± 1.7 a	55.6 ± 1.5 a	49.4 ± 1.8 a	7.57 ± 0.07 a	8.06 ± 0.03 a	7.53 ± 0.12 a	0.46 a	0.42 a	0.46 a
	S1	287 ± 3 b	288 ± 6 b	265 ± 4 b	30.5 ± 2.7 b	30.9 ± 2.2 b	33.1 ± 0.3 b	37.6 ± 0.5 c	38.6 ± 0.5 c	37.8 ± 1.8 c	3.32 ± 0.02 c	3.25 ± 0.13 c	3.32 ± 0.07 c	0.29 c	0.27 c	0.28 c
	S2	323 ± 7 a	324 ± 4 a	312 ± 7 a	42.9 ± 1.5 a	43.2 ± 1.5 a	46.1 ± 0.7 a	47.9 ± 1.9 b	48.0 ± 1.6 b	42.7 ± 1.4 b	6.62 ± 0.08 b	6.71 ± 0.18 b	6.14 ± 0.04 b	0.40 b	0.37 b	0.38 b
CM-34	CK	324 ± 6 a	325 ± 6 a	390 ± 4 a	45.0 ± 1.8 a	45.2 ± 1.8 a	34.9 ± 0.6 a	55.6 ± 0.1 a	54.8 ± 2.3 a	53.1 ± 0.9 a	8.38 ± 0.19 a	8.16 ± 0.13 a	7.23 ± 0.04 a	0.46 a	0.43 a	0.44 a
	S1	287 ± 6 b	288 ± 6 b	299 ± 10 b	29.2 ± 3.4 b	28.3 ± 0.5 b	30.3 ± 1.4 b	34.7 ± 0.4 c	36.8 ± 0.5 c	35.2 ± 0.7 c	3.10 ± 0.17 c	2.99 ± 0.09 c	3.19 ± 0.03 c	0.27 c	0.26 c	0.28 c
	S2	326 ± 7 a	327 ± 7 a	390 ± 8 a	44.2 ± 1.8 a	44.8 ± 0.4 a	33.0 ± 0.7 a	44.9 ± 0.6 b	48.1 ± 1.1 b	46.6 ± 0.8 b	6.72 ± 0.13 b	7.03 ± 0.03 b	6.00 ± 0.11 b	0.39 b	0.37 b	0.38 b
Source of variance																
Cultivars (C)		13 ^**^	10 ^**^	13 ^**^	173 ^**^	146 ^**^	169 ^**^	100 ^**^	30 ^**^	42 ^**^	1196 ^**^	769 ^**^	963 ^**^	245 ^**^	257 ^**^	118 ^**^
Shading (S)		803 ^**^	720 ^**^	810 ^**^	684 ^**^	685 ^**^	690 ^**^	711 ^**^	476 ^**^	668 ^**^	6366 ^**^	6755 ^**^	5700 ^**^	205 ^**^	359 ^**^	209 ^**^
C×S		22 ^**^	20 ^**^	22 ^**^	3 ^*^	6 ^**^	7 ^**^	13 ^**^	1 ^*^	5 ^**^	110 ^**^	84 ^**^	79 ^**^	47 ^**^	66 ^**^	11 ^**^

Different letters indicate statistically significant differences at the levels of 0.05. ^∗^ *P*< 0.05; ^∗∗^ *P*< 0.01.

Pre- and post-anthesis shading decreased the HI by 34.1%–41.2%, and 10.1%–14.1%, respectively, compared to that of plants in the no-shading plots (**Table 3**). A portable reason is that shading stress increased the stem-to-leaf ratio and decreased the grain-to-glume ratio (**Figure 10**). The HI for the shade-sensitive cultivars (SM-482 and CM-34) decreased more rapidly compared with those for the shade-tolerant cultivars (CM-39 and MM-51).

## 4 Discussion

### 4.1 Shading decreases *P*_N_ through Rubisco-mediated non-stomatal limitation

The speed of photosynthetic adjustment to changing light environments strongly affects daily carbon gain and grain yield (Mathur et al., 2018). Considering that plants respond differently to canopy light intensity for both shaded and unshaded plants, the changes in *g*_S_ and *L*s contributing to *P*_N_ reduction in changing canopy light intensity are more critical for uncovering the underlying mechanisms for variations in plant biomass and grain yield. Our results agree with previous results showed that the *P*_N_ was reduced when plants were exposed to shade stress (Chen et al., 2017; Wang et al., 2020b). Notably, we found that shading had two distinct effects on leaf photosynthesis. First, shading decreased the photosynthetic capacity of flag leaves owing to Rubisco-mediated non-stromal limitations. This conclusion was supported by evidence showing that Rubisco activity, *g*_S_, *L*s, and *T*r of flag leaves decreased with decreasing light intensity, whereas *C*_i_ increased. A decrease in *L_S_* indicated a decrease in the resistance of CO_2_diffusion to the intercellular space. An increase in *C*_i_ indicated that plants in struggling to utilize intercellular CO_2_ effectively due to insufficient light energy. Rubisco is a crucial enzyme responsible for fixing CO_2_ into organic compounds (Bambach and Gilbert, 2020). Maintaining high Rubisco activity is critical for increasing daily carbon gain in shaded environments. Rubisco reduction is known to be a rapid response of leaves to shade stress, and the reduction of the Rubisco carboxylation rate can be compensated by rapid activation (Gao et al., 2020a; Zhao et al., 2022). By downregulating Rubisco, plants can redirect resources to other metabolic processes that are more critical for survival under stressful environments (Taylor et al., 2022). This finding explained that the shade-tolerant cultivar CM-39 showed a lower reduction rate of Rubisco activity in shaded environments than that of the shade sensitivity cultivars CM-34. Shading perturbs CO_2_ assimilation and decreases daily carbon gain, as evidenced by the strong correlation between CE and RuBisCO activity in normal and shaded environments. The PEPC plays a crucial role in converting fixed carbon into organic acids, its reduced activity confirmed that wheat plants struggle to produce the necessary photosynthetic products for plant growth and grain filling. Results in soybean validated the critical role of PEPC in shade tolerance through weighted gene co-expression network analysis (Jiang et al., 2023). Notably, *g*_S_ decreased rapidly when the canopy light intensity was lower than 400 μmol m^-2^ s^-1^, indicating that only extremely weak light can increase the the resistance of CO_2_diffusion to the intercellular space. A novel finding is that WUE_i_ decreased with PAR, and a greater WUE_i_ decline was observed for the shade-sensitivity cultivar (CM-34) than the shade-tolerant cultivar (CM-39). A reasonable explanation is that the speed of stomatal response to light intensity cannot keep up with the speed of *P*_N_ response to light intensity, causing a decrease in the exchange efficiency of stomatal H_2_O and CO_2_. These results indicated that shading decreased photosynthesis primarily by Rubisco-mediated non-stomatal limitation and secondarily by reducing *g*_S_.

### 4.2 Shade-tolerant cultivars adapted to low-light conditions due to morphological and physiological acclimations

Our results showed that wheat plants adapt to low-light conditions and show a high grain yield due to morphological and physiological acclimations; this maximizes the solar energy conversion efficiency. Previous results showed that increased stem elongation is a typical shade avoidance characteristic, enabling plants to maximize light interception (Chen et al., 2020; Zhong et al., 2020). However, only a few studies have focused on leaf-level acclimation. When light is limited, the adaptive response to increase the solar energy conversion efficiency in leaves is to increase the single leaf area (Arenas-Corraliza et al., 2019). In this study, we found that shading increased the leaf length of flag leaves but decreased the LAI. A possible reason could be that a high singal leaf area enables plants to capture more solar energy in low-light environments (Hussain et al., 2019). We also found that shading decreased LMA, which enabled plants to intercept more solar energy in low-light environments (Wu et al., 2017a). LMA is negatively related to light availability (Poorter et al., 2009) and leaf thickness (Dong et al., 2019). A positive consequence of decreased LMA is the increased CO_2_ diffusion from the atmosphere to leaves, as evidenced by the rapid decrease in the *g*_S_ and CE when canopy light intensity was less than 400 μmol m^-2^ s^-1^.

At the physiological level, we found that shading increased chlorophyll *a* and *b* contents but decreased the chlorophyll *a*/chlorophyll *b* ratio, indicating a typical vegetative response of acclimation to low-light conditions. In this way, plants exposed to shading can extend the useful wavelength range to a shorter wavelength, improving the light absorption ability of the chloroplast, which eventually help in utilizing CO_2_ levels as photosynthesis becomes more efficient (Wu et al., 2017b; Zhao et al., 2022). Shading increased chlorophyll *a* and *b* contents in flag leaves, which can be attributed to carbohydrate metabolism decreasing more rapidly than nitrogen metabolism in low-light environments. However, the increase in chlorophyll *a* under shaded conditions was insufficient to compensate for the decreased *P*_N_ caused by the decreasing light intensity. Therefore, shade-tolerant cultivars adapted to low-light conditions mainly by increasing the leaf area of flag leaves to capture solar energy and by increasing the chlorophyll levels to convert solar energy to chemical energy.

### 4.3 Shading affected the source and sink relationship and morphological traits of the endosperm

Reductions in grain weight and yield due to abiotic stress during the grain-filling stage are associated with the photosynthetic capacity of flag leaves and sucrose availability in developing grains (Ishibashi et al., 2014; Yang et al., 2023). In the present study, shading decreased sucrose content in both flag leaves and developing grains, which was more evident at 14–28 DAA. These results indicate that shading decreases grain weight by reducing *P*_N_ and sucrose availability in flag leaves, because the remobilization of carbohydrates from the leaf to developing grains determines the grain weight and yield of most cereal crops. We also observed that the effect of shading on sucrose content was more significant in the flag leaves than in the developing grains. This might be attributed to the buffering effect of the stored carbohydrates in the leaves and stems. Pre-anthesis shading decreased the sucrose content and grain weight of developing grains. This finding supports our conclusion that shade stress impairs the photosynthetic system of flag leaves and that light restoration after anthesis cannot compensate for the loss in photosynthetic capacity. Another study showed that abiotic stress during the grain-filling stage increased seed abortion, resulting in compensatory increased growth of the remaining kernels (Shen et al., 2020). In the present study, we observed a compensatory increase in grain weight but not in grain sugar content. This might be because the compensatory effect of seed abortion is much lower than the amount of sucrose remobilized from the source to sink organs. We highlight the importance of sucrose in regulating the **“**live or die**”** choice of kernels and grain weight under shade stress.

Results observed in cotton (Hu et al., 2016) and maize (Wang et al., 2021a) indicate that shading reduces the biosynthesis and recycling of sucrose. In the present study, we observed that sucrose metabolic enzymes in both flag leaves and developing grains were dramatically affected by shading treatments, indicating that sucrose metabolic enzymes contributed to grain-filling. In developing grains, sucrose is degraded to hexoses via SuSy, which provides substrates for starch biosynthesis and grain-filling. Thus, shading affected the grain weight and uniformity of starch granules by reducing sucrose degradation enzymes in developing grains. Shading decreased the activity of SuSy in flag leaves to a greater extent than that in developing grains, resulting in a decreased source-to-sink ratio and, ultimately, decreased grain-filling rate and grain yield. We also observed that sucrose biosynthesis in developing grains increased owing to shading treatments and increased SPS activity, adversely affecting starch biosynthesis and grain filling. Shading reduced carbohydrate transformation into starch and thus decreased the 1,000-kernel weight and grain yield. Further studies are required to analyze the genetic variation of critical shade tolerance traits in a historical set of cultivars, which will help to identify critical traits that increase solar energy conversion efficiency in low-light environments.

### 4.4 Yield loss by shading stress depending on the shading period and cultivar plasticity

Biomass was closely related to the number of fertile tillers (R^2^ = 0.66), demonstrating that the reduction in the number of tillers by shading reduced the capacity of intercept radiation and utilization of photosynthetic active radiation, and thereby penalizing biomass and yield. as evidenced by previous studies on wheat (Qiao et al., 2019; Wang et al., 2020b) and rice (Song et al., 2022; Wang et al., 2015). The novel finding in the present study is that the yield loss caused by shading treatments varies depending on cultivar plasticity and shading period. Yield loss resulting from pre-anthesis shading was higher than post-anthesis shading. This finding can be attributed to the decreased intercepted PAR under pre-anthesis shading being higher than post-anthesis shading (Yang et al., 2023). Our study found that post-anthesis shading decreased grain yield, mainly by decreasing 1,000-kernel weight, similar to the results of maize shaded during the post-silking stage (Shen et al., 2020; Wang et al., 2020a, Wang et al., 2021a; Yang et al., 2016). Comparatively, pre-anthesis shading decreased the grain yield by decreasing the grain number per spike and 1,000-kernel weight. Pre- and post-anthesis shading alters the source-to-sink ratio. As a result, the grain number per spike and 1,000-kernel weight were reduced, reducing plant biomass and grain yield. The decreased grain number per spike can be attributed to insufficient carbohydrates increasing the competition between kernels, resulting in increased insemination and seed abortion (Deng et al., 2021; Dong et al., 2019; Ren et al., 2022). Previous results showed that yield reduction due to pre-anthesis shading could be compensated for by an increase in 1,000-kernel weight in the later stages of wheat growth (Labra et al., 2017; Song et al., 2022). In the present study, pre-anthesis shading decreased both grain number per spike and 1,000-kernel weight, indicating that light restoration after anthesis could not fully compensate for the damage to the photosynthetic system caused by low-light conditions. Therefore, yield loss due to low-light conditions depends on the shading period and cultivar plasticity.

The selection of shade-tolerant cultivars provides a promising approach to reducing yield loss from low-light conditions. We found that the maximum yield loss due to shading was more evident by weak gluten cultivars than that strong gluten cultivars. A notable finding is that cultivars (CM-34) bred in the strong-light ecological regions were more sensitive to weak-light conditions than those bred in the low-light regions. A possible explanation is that the reductions in Rubisco activity, *P*_N_, and *g*_S_ of CM-34 under low-light conditions were higher than the reductions in those of other cultivars. Shading decreases the diffusion of CO_2_ into leaves and CO_2_ assimilation, thus decreasing the photosynthetic capacity of wheat flag leaves and ultimately decreasing plant biomass and grain yield. The grain yield of wheat mainly depends on post-anthesis photosynthesis, and carbohydrates are remobilized from vegetative organs to developing grains during the post-anthesis period. The current study found that shading increased the retention of assimilates in vegetative organs, leading to insufficient carbohydrates for grain filling, which ultimately decreased HI and grain yield. The decreased HI might be due to the decreased expression of the sucrose transport gene SUT1 (Ishibashi et al., 2014). Shading decreased the photosynthetic capacity of flag leaves and the assimilate partitioning to grains, resulting in decreased sucrose content in developing grains, which ultimately decreased grain-filling rate and grain yield. Shade-tolerant cultivars adapted to shade stress showed high grain yield by increasing single leaf area and chlorophyll content, resulting in increased light harvesting potential, ultimately reducing yield loss in the context of global dimming.

## 5 Conclusion

Yield loss by shade stress varied depending on cultivar plasticity and shading period. Pre-anthesis shading decreased grain yield (13.1%–42.9%) mainly by decreasing grain number per spike and 1000-grain weight. In contrast, post-anthesis shading decreased grain yield (38.6%–61.2%) mainly by decreasing the 1,000-grain weight. Pre-anthesis shading impairs the photosynthetic system; restoring light intensity after anthesis cannot fully compensate for the decreased grain filling rate and 1,000-grain weight. Yield loss due to shade stress was due to Rubisco-mediated non-stomatal limitations and secondarily due to reducing g_S_ in flag leaves. Shade-tolerant cultivars adapted to low-light conditions and showed a lower yield loss by increasing leaf length of flag leaves, chlorophyll content, and LMA for higher light harvesting. Our study provides information for uncovering the mechanisms underlying shade stress tolerance and will help design strategies to reduce yield loss in low-light environments in the future. Further studies are required to identify key genes and regulatory networks involved in photosynthetic limitations and acclimations, which will provide valuable insights for reducing yield loss in the context of global dimming.

## Data Availability

The original contributions presented in the study are included in the article/supplementary material. Further inquiries can be directed to the corresponding authors.
